# Modelling the Impact of Vector Control on Lymphatic Filariasis Programs: Current Approaches and Limitations

**DOI:** 10.1093/cid/ciab191

**Published:** 2021-06-14

**Authors:** E L Davis, J Prada, L J Reimer, T D Hollingsworth

**Affiliations:** 1 Big Data Institute, University of Oxford, Oxford, UK; 2 University of Surrey, Guildford,UK; 3 Liverpool School of Tropical Medicine, Liverpool,UK

**Keywords:** lymphatic filariasis, vector control, modelling, elimination, resurgence

## Abstract

Vector control is widely considered an important tool for lymphatic filariasis (LF) elimination but is not usually included in program budgets and has often been secondary to other policy questions in modelling studies. Evidence from the field demonstrates that vector control can have a large impact on program outcomes and even halt transmission entirely, but implementation is expensive. Models of LF have the potential to inform where and when resources should be focused, but often simplify vector dynamics and focus on capturing human prevalence trends, making them comparatively ill-designed for direct analysis of vector control measures. We review the recent modelling literature and present additional results using a well-established model, highlighting areas of agreement between model predictions and field evidence, and discussing the possible determinants of existing disagreements. We conclude that there are likely to be long-term benefits of vector control, both on accelerating programs and preventing resurgence.

Lymphatic filariasis (LF) is a filarial worm infection transmitted by mosquitoes that can lead to permanent and debilitating disability if left untreated. Almost 900 million people are at risk of infection worldwide and the disease has been targeted for elimination as a public health problem (EPHP) by 2030 by the World Health Organization (WHO) [1]. EPHP is an operational definition, associated with a population microfilaria prevalence of 1% or antigenemia prevalence of 2% in the majority of settings, and is validated using specifically designed Transmission Assessment Surveys (TAS) [1]. EPHP validation results in a switch of program focus to post-validation surveillance (PVS). To date, 16 countries and territories worldwide have been acknowledged as achieving EPHP, with preventative chemotherapy still required in 49 countries [1].

For many vector-borne diseases, such as malaria, substantial gains have been achieved using vector control [2,3], however in LF programs, mosquito control is only considered as a supplemental measure [1]. The WHO recommended strategy for reducing transmission of LF is a minimum of 5 years of annual mass drug administration (MDA) at 65% coverage in the majority of settings. Treatment is usually a combination of diethylcarbamazine and albendazole (denoted DA), or ivermectin and albendazole (IA), but recent evidence has led to the WHO also recommending the use of all 3drugs (IDA) in certain areas that are not co-endemic with onchocerciasis or loiasis, such as the Indian subcontinent [1]. Current WHO guidance supports use of long-lasting insecticide-treated nets (LLINs) in areas where *Anopheles* is the primary vector to reduce transmission, but it is not required for EPHP validation either during or after cessation of the MDA program.

The target of EPHP was originally defined as a stepping-stone towards elimination of transmission and, eventually, global eradication [4], but a number of modelling studies have suggested that a lower threshold would be required before MDA cessation in the majority of settings if the end goal is elimination of transmission [5–7]. True elimination is dependent on a transmission breakpoint—the threshold prevalence below which numbers should naturally decline to zero [8].

The existence of a breakpoint is derived from attrition at each stage of the parasite’s life cycle, no replication within the vector, and the requirement for sexual reproduction in the human host; male and female adult worms are required in the same host for male-female (mf) production, and therefore infectiousness [8]. The breakpoint is the threshold prevalence at which the likelihood of sexual reproduction in a host drops sufficiently low that sustained transmission is no longer viable. This breakpoint depends on a number of factors, including the mosquito biting rate, and reductions in biting increase the breakpoint. The threshold biting rate is the lowest biting rate at which transmission can be sustained; if it is reduced below this level, then case numbers will decline to zero no matter how high the starting prevalence is [5].

Mathematical models of transmission have been previously used to derive estimates of breakpoints and thresholds for elimination [4, 5, 9] and have the potential to help inform where and when resources, such as vector control, would be best targeted. However, vector control is often a secondary consideration and current models are not necessarily designed with analyzing vector-based interventions in mind. In this article, we review the current field evidence and modelling literature on vector control usage for lymphatic filariasis control and elimination and discuss how modelling methods could be extended to more accurately capture vector dynamics.

## EXISTING EVIDENCE

### Field Evidence

There is strong global evidence that vector control, including bednets, environmental management, and indoor residual spraying, greatly reduces filariasis prevalence and, in many instances, the effect is greater for filariasis than for other vector-borne diseases [10]. The impact of bednets on filariasis transmission far exceeds the impact on reducing mosquito biting densities; a study in Kenya showed a 22% reduction in biting rates but a 95% reduction in the annual infective biting rate [11]. Another study in Papua New Guinea (PNG) reported a decrease from 61 bites per person per day to 9, and a reduction in the annual infective biting rate (AIBR) to zero following the distribution of LLINs [12].

Some of the strongest evidence of the role of bednets in filariasis elimination is the case of the Gambia, where elimination occurred during the scale up of insecticide-treated nets, and in the absence of any MDA [13]. Bednets not only provide personal and community protection against infective bites, they reduce the likelihood of daily survival in mosquitoes, and thus the proportion of the population that survives through the extrinsic incubation period.

Vector control was the primary control strategy for filariasis before the switch to mass drug administration of preventive chemotherapy [10]. There has been renewed enthusiasm to include vector control in the Global Programme to Eliminate Lymphatic Filariasis (GPELF) due to challenges with achieving the scale, coverage and continuity of MDA [14].

### Recent Modelling

To review the recent modelling literature, we searched PubMed using the search term “vector control” and “lymphatic filariasis” and either “model,” “modelling” or “dynamics” on 22 October 2020, for articles published in the last 10 years (since 2010). The search returned 30 publications, from which we identified 10 that used mathematical models of lymphatic filariasis transmission to assess the impact of vector control on program outcomes [2, 5, 6, 12, 15–20]. Of these studies, 4 used reported vector control coverage data from specific settings and 6 considered a theoretical introduction of vector control at specified coverage levels, most commonly 50% or 80% population coverage. The majority (7 studies) considered LLINs only, 2 considered LLINs or larval control, and 1 considered a generic impact of vector control, interpreted as LLINs or IRS. Eight studies considered vector control in combination with MDA, with a range of drug regimens (IA, DA, DEC salt and/or IDA), and 2 considered the impact of vector control in isolation.

There were 3 models that appeared multiple times across these studies [5–7, 15–18, 20]. Two of these were stochastic individual-based models that track the unique infection status of individuals within a population, and the third was a deterministic age-structured model that tracks the mean population burden of worms and infective mf stages. However, all 3 models consider the infection dynamics in vectors as deterministic and implement vector control as a reduction in the overall biting rate.

TRANSFIL, one of these stochastic individual-based models, has been fitted to historical data using Approximate Bayesian Computation methods and also considers systematic noncompliance of MDA [17,18]. Bednets are modelled to have 2 separate impacts on transmission: they reduce the transmission from the host population to the vector population, and they reduce the infective biting rate. Individual hosts are modelled distinctly as either protected by a net or not, according to a binomial probability equal to assumed coverage, with those who are protected experiencing a lower bite rate. However, the impact on the infective larval population in the vector is calculated using a deterministic mean-field approach, assuming that net coverage correlates to a proportional mean reduction in larval uptake across the vector population.

Results using this model are varied, with one study claiming that 50% vector control may only have a small impact on number of rounds of the triple-drug (IDA) required to reach the 1% threshold, with no observable difference at low prevalence (10% mf) and approximately one round of difference at high prevalence (40% mf) [20]. However, a later study focusing on PNG, which used a combination of modelling and field data to consider variable heterogeneity in mosquito-bite exposure and infection distributions, found that use of bednets could rapidly reduce the number of rounds required [18], aligning better with the existing field evidence. These differences could be due to contrasting assumptions around systematic nonadherence or that the second study more carefully considers the relationship between host and vector heterogeneities. An additional study found that 50% vector control coverage can result in large increases in elimination probability—from 3% to 97% in some *Anopheles* settings [17].

The other stochastic model is LYMFASIM, which has similar host dynamics but uses a deterministic nonlinear model for the infection dynamics in the vector. The infection level in the vector population is derived from the individual mf density and individual exposure to mosquito bites across all hosts and is governed by a nonlinear relationship between mf intensity in the human blood and the development and survival rates of infective stage (L3) larvae in mosquitoes [21]. Vector control is modelled using similar methods to TRANSFIL, assuming a reduction in the biting rate proportional to coverage of the intervention.

In a recent study, LYMFASIM predicts implementing 50% coverage vector control will lead to no change in the median number of rounds to achieve the EPHP threshold in lower prevalence (10% mf) settings, but results do show a reduction in variability between outcomes [20]. The same study shows approximately a one-round improvement for higher prevalence settings (40% mf), similar to TRANSFIL, but a larger decrease in the range of rounds required.

The third model, EPIFIL, is deterministic and uses age-structured partial difference equations to describe the mean worm burden and mf levels in humans and an ordinary differential equation to describe the infective L3 density in the vector population [19]. LLIN usage is considered to reduce the vector to host ratio according to 3 parameters estimating the deterrence, feeding inhibition, and toxicity of insecticides [16]. MDA coverage is assumed to be random, with no ability within the model to account for systematic nonadherence.

In general, studies using EPIFIL focused on calculating the mf breakpoint and the timelines and probabilities for true elimination, with broad conclusions that vector control can have a substantial impact on these outcome measures [5, 16, 19]. One study showed that using 80% coverage vector control to increase the mf breakpoint could reduce the number of MDA rounds required to ensure a 95% probability of elimination by 6 to 15 rounds, with a median decrease of 9 rounds, and could reduce the variance from 3.52 to 2.66 in *Anopheles* settings [16]. A recent study using vector control and infection data from a range of settings demonstrated that maintaining vector control at current levels (25%–80%) after achieving 1% mf prevalence gave an elimination probability of 91%–100%, whereas the one setting investigated that had no vector control had a much lower elimination probability of 24% [15].

However, other studies suggest only a small impact of increasing LLIN coverage to 80% on elimination year and no discernible difference on rounds of IDA treatment to 1% mf prevalence between no vector control and 50% vector control for either low or high prevalence settings [6, 20]. Modelling also found only very minor gains from increasing vector control coverage from 50% to 80% on additional rounds required to get from 1% mf to the actual breakpoint required for true elimination [7]. This may be due to the lack of systematic nonadherence in the model, resulting in an overestimation in the impact of MDA on prevalence, which may give a lower comparative effect of vector control.

The other 2 studies used different modelling assumptions. A data-driven study based in PNG that used xeno-monitoring methods to quantify the prevalence of infection, and infectiousness in the vector population found no infectious vectors following LLIN distribution and used Bayesian modelling methods to conclude that transmission had been interrupted in all locations [12]. The second study used a simple differential equation model to consider the worm burden in the human population and concluded that relatively modest vector control coverage (36%) could lead to LF elimination without any requirement for MDA [2], which supports findings from The Gambia [13].

## METHODS

We used one of the previously discussed models of LF transmission, TRANSFIL, a stochastic individual-based model that captures the basic processes relevant to the transmission of LF, including vector density and biting rate, parasite life cycle, and human exposure to vectors [17, 22]. A proportion of the host population, equal to the chosen vector control coverage, are considered to be protected by either LLINs or IRS and therefore experience reduced transmission of infective larvae from mosquitoes. Uptake of new infectious larval infections in the vector population is considered to be reduced in proportion to modelled vector control coverage. The annual biting rate in the absence of vector control was varied between 0 and 1200 bites per person per year and the aggregation parameter for bite risk, k, between 0.01 and 0.1.

Our results focus on moderate mf prevalence (10% ± 1%) settings with *Anopheles* as the dominant vector, as these are the areas where vector control is expected to have most utility. Annual MDA using IA is simulated every 12 months at 65% coverage until mf prevalence is below 1% for a full year post-MDA, at which point MDA is halted and the model is then run for a further 10 years. Systematic nonadherence to MDA is included in the model by calculating individual probabilities of receiving MDA based on coverage and a between-round correlation parameter [23].

To investigate the impact of vector control on different stages of LF elimination, we simulated 4 main combinations of this MDA regimen and different vector control strategies: 1. No vector control (counterfactual); 2. 50% vector control coverage during MDA, sustained at same levels post-EPHP; 3. 50% vector control coverage during MDA, enhanced to 80% coverage post-EPHP; 4. 50% vector control coverage during MDA but no maintenance of vector control post-EPHP, leading to waning effects on transmission (see Supplementary Materials). The primary vector control method considered was LLINs and insecticide waning effects were modelled assuming a 2-year half-life [24].

Model outcomes were categorized using a Pearson’s correlation test between time and the mf prevalence across the first 9 years following MDA cessation; if correlation was significant (*P* = .01) then a positive correlation coefficient was taken to represent increasing prevalence (“resurgence”) and a negative correlation to represent prevalence decreasing towards zero (“true elimination”). In a small proportion of scenarios correlation was found to be not significant (*P* > .01) and this was interpreted as transmission persisting at a low-level with no evidence of resurgence or elimination (“low-level maintenance”).

## RESULTS

**Figure 1.**
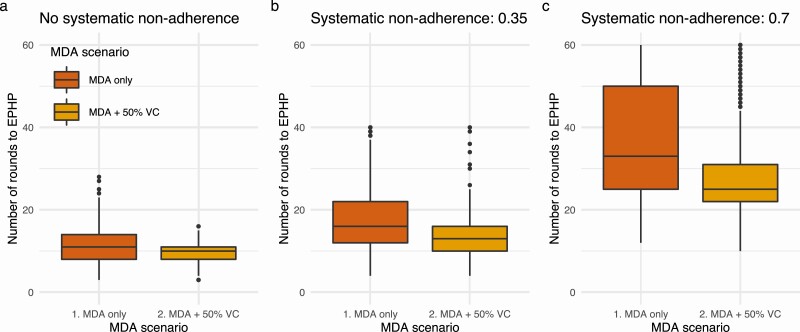
Modelled impact of vector control on MDA rounds to EPHP. The number of rounds of MDA (65% IA, *Anopheles* settings) required to reach EPHP (1% mf prevalence) from a baseline prevalence of 9%–11% (aggregation k from 0.01 to 0.1 and ABR from 0 to 1200). MDA only (red) and MDA with 50% vector control coverage (orange) and a range of assumptions around systematic nonadherence: (*A*) No systematic nonadherence; (*B*) moderate systematic nonadherence (correlation 0.35); (C) high systematic nonadherence (correlation 0.7).

The impact of vector control on the number of MDA rounds required to reach EPHP levels is dependent on assumptions about systematic nonadherence to MDA (Figure 1). Implementing 50% coverage vector control alongside MDA, assuming no systematic nonadherence, reduces the median number of rounds from 11 to 10 and the mean from 11 to 9 rounds. Although this is a comparatively small average impact, vector control does have a substantial effect on variability in the number of rounds required to achieve EPHP, with a reduction in the IQ range from 8–14 to 8–11 and a reduction in the upper 95% quantile from 18 to 13 rounds.

For systematic nonadherence correlation of 0.35, 50% vector control gives a difference of 3 rounds between the median number of rounds to reach the EPHP threshold: 16 (IQR: 12–22) for MDA only, 13 (IQR: 10–16) for MDA and 50% vector control. Assuming a very high level of systematic nonadherence correlation, 0.7, implementing 50% coverage vector control alongside MDA reduces the median from 33 (IQR: 25–50) to 25 (IQR: 22–31), a saving of 8 rounds in the median scenario.

**Figure 2.**
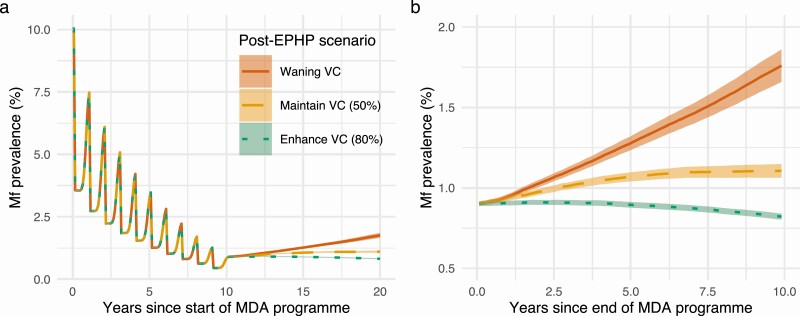
Modelled impact of vector control on elimination and resurgence trajectories. Mean mf prevalence for scenarios with 65% coverage of IA and 50% coverage vector control during MDA (*Anopheles* settings, 9%–11% baseline mf prevalence) that reach EPHP in 10 rounds. Following MDA cessation, 3 scenarios are considered: waning vector control efficacy due to poor or no maintenance (red, solid); vector control maintained consistently at 50% coverage (orange, dashed); enhanced 80% coverage vector control (green, dotted).

Looking at mf prevalence over time during and post-MDA shows clear trends between vector control usage and long-term dynamics of lymphatic filariasis transmission (Figure 2). Enhancing vector control after MDA cessation can reduce the risk of resurgence and keep prevalence at low levels. However, vector control used during an MDA program that is not maintained following EPHP validation could accelerate resurgence.

**Figure 3.**
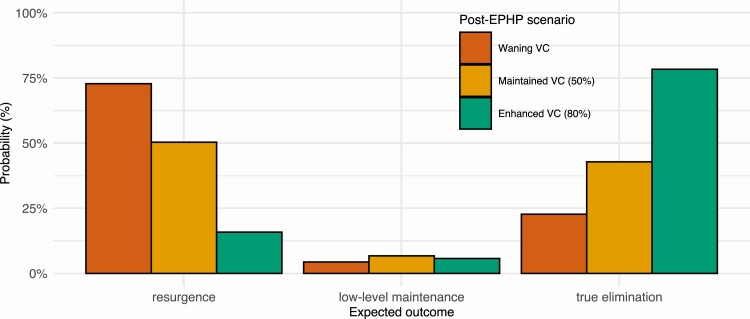
Modelled impact of vector control on probabilities of elimination and resurgence. Probability of resurgence (left), low-level maintenance (center), and true elimination (right) following EPHP validation and MDA cessation for *Anopheles* settings with a 9%–11% baseline prevalence. Following MDA cessation, 3 scenarios are considered: waning vector control efficacy due to poor or no maintenance (red, left); vector control maintained consistently at 50% coverage (orange, center); enhanced 80% coverage vector control (green, right).

After MDA cessation, vector control usage has a substantial impact on long-term outcomes (Figure 3). In the model maintaining 50% coverage, vector control after reaching EPHP prevalence levels and stopping MDA reduced the risk of resurgence (increasing transmission) from 72.9% to 50.4% and more than doubled the probability of true elimination (decreasing transmission towards zero) from 22.8% to 42.9%. Enhancing coverage from 50% to 80% also had a large benefit, reducing the risk of resurgence to 15.9% and increasing the probability of elimination to 78.4%.

## Discussion

We found a number of areas of agreement between modelling and field evidence for vector control. In particular, reports of vector control being successfully used to interrupt transmission in the field [12, 13] are supported by model predictions that reducing the biting rate could greatly increase the probability of elimination and decrease the probability of resurgence [2, 15, 17]. Our results also demonstrate the utility of enhancing vector control coverage after MDA cessation and that poorly maintained vector control could undermine hard-won gains.

However, modelling studies are variable in their assessment of the predicted impact of vector control on the required duration of MDA programs to achieve a threshold of 1% mf prevalence. A number of studies have concluded a limited reduction of up to 1 round for 50% vector control coverage [6, 20], but these findings are not consistent across all recent modelling [18]. We have demonstrated that this inconsistency could be partially due to the choice of key parameters, such as the level of systematic nonadherence. Lower systematic nonadherence results in more effective MDA interventions and a lower relative impact of vector control on number of rounds to the threshold, whereas high systematic non-adherence is associated with a higher relative impact of vector control.

Current modelling methods are limited by largely simplified assumptions around the vector dynamics, potentially resulting in an underestimation of vector control impact. Most models assume vector control provides a reduction in biting, either on average across the population or for individual protected hosts, and a reduced overall uptake in mf by the vector population [20]. However, none of the models discussed consider the age-structure of the vector population, or how this changes under the pressures of vector control. For example, LLIN usage deters biting and increases mortality in blood-seeking mosquitoes, which will translate to a younger vector population that each take fewer successful blood meals across their lifespan. As a result, fewer infected vectors would survive the extrinsic incubation period to infectivity, which is not captured in current modelling methods and could explain some of the discrepancies between model predictions and the field evidence.

Models are also currently not generally well-validated for low prevalence, but we know the qualitative dynamics are able to emulate a range of scenarios. In particular, modelling has demonstrated that there is a long tail to elimination [4], supporting observations of low-level maintenance in countries that have achieved EPHP [25], and agrees with findings that vector control can substantially reduce the length of this tail [6, 7].

Despite overall agreement that vector control has demonstrable benefits for LF control and elimination, the areas of difference we have discussed between the field evidence and modelling results are indicative of how modelling methods could be built on in the future to better address the utility of vector control. As more countries approach EPHP validation, there is a greater need than ever to understand the determinants of elimination. Explicit inclusion of vector population structure and dynamics in models of LF transmission would enable more focused and detailed analysis into how, where, and when vector control resources are best directed.

## Conclusions

Although models and field data currently provide conflicting messages on the magnitude of any potential impact of vector control during MDA, there is a strong agreement between the modelling literature and the field evidence that vector control is highly beneficial post-MDA in reducing resurgence and increasing the probability of elimination of transmission. We conclude that it is vital that existing vector control interventions are well-maintained after MDA cessation, and that there is likely to be substantial long-term benefit to implementing enhanced vector control coverage alongside post-validation surveillance activities.

## Supplementary Data

Supplementary materials are available at *Clinical Infectious Diseases* online. Consisting of data provided by the authors to benefit the reader, the posted materials are not copyedited and are the sole responsibility of the authors, so questions or comments should be addressed to the corresponding author.

ciab191_suppl_Supplementary-Material

## Nonstandard Abbreviations:

AIBR: annual infective biting rate
DA: DeC and albendazole
DEC: diethylcarbamazine
IA: ivermectin and albendazole
EPHP: elimination as a public helath problem
GPELF: Global Programme to Eliminate Lymphatic Filariasis
IDA: ivermectin
DEC: and albendazole
IQR: interquartile range
IRS: indoor residual spraying
LF: lymphatic filariasis
LLINs: long-lasting insecticide-treated nets
MDA: mass drug administration
mf: male–female
PNG: Papua New Guinea
PVS: post-validation surveillance
WHO: World Health Organization

## Notes

***Supplement sponsorship.*** This article appears as part of the supplement “Sustainable control of neglected tropical diseases – surveillance and diagnostics”, sponsored by the NTD Modelling Consortium.

***Disclaimer.*** The views, opinions, assumptions, or any other information set out in this article are solely those of the authors and should not be attributed to the funders or any other person connected with the funders.

***Financial support.*** This work was supported by The Bill & Melinda Gates Foundation [grant number OPP1184344].

***Potential conflicts of interest.*** E. D. and J. P. report grants from the NTD Modelling Consortium. All authors: No reported conflicts of interest. All authors have submitted the ICMJE Form for Disclosure of Potential Conflicts of Interest. Conflicts that the editors consider relevant to the content of the manuscript have been disclosed.
