# Supplementary material for: Modelling the Impact of Vector Control on Lymphatic Filariasis Programs: Current Approaches and Limitations
Source: Clin Infect Dis. 2021 Jun 14;72(Suppl 3):S152–7. doi: 10.1093/cid/ciab191 (PMC8201547; doi:10.1093/cid/ciab191)
Supplement: ciab191_suppl_Supplementary-Material [file ciab191_suppl_supplementary-material.docx]

**Supplementary Information**

**Table I:** Brief summary of modelling papers found in literature search: selected from a search of PubMed using the search terms "vector control" and "lymphatic filariasis" and either "model", “modelling” or “dynamics” on 22^nd^ October 2020, for papers published in the last 10 years (since 2010).

| **Paper** | **Continent(s) and/or countries** | **MDA** | **Bednet coverage**  **(**baseline**, *enhanced*)** | **Impact of increasing vector control from** baseline **to *enhanced*** |
| --- | --- | --- | --- | --- |
| Reimer et al. (2013) | PNG | none | 0-12%, ***75-91%*** (data) | Cut transmission potential to zero infective vectors |
| Stone et al. (2014) | Non-specific | none | 0-100% | Vector control alone could lead to elimination of transmission. |
| Irvine et al. (2015) | Africa, Asia | DA, IA | 0%, ***50%*** | Increases elimination probability (3 to 97%) |
| Singh et al. (2015) | Africa, Asia, PNG | DA, IA | 0%, ***80%*** | Increases mf prevalence ‘breakpoint’ |
| Michael et al. (2016) | Africa, Asia, PNG | DA, IA | 0%, ***80%*** | Fewer MDA rounds required (6-20 to 2-13) |
| Michael et al. (2017) | Africa | DA | 11-91% (data), ***80%*** | Minor impact on elimination year |
| Smith et al. (2017) | Africa, Asia, Haiti | DEC salt, DA, IDA | 0%, ***50%, 80%*** | Fewer MDA rounds required (mean: 3.6 fewer) but little benefit from 50% to 80% |
| Irvine et al. (2017) | Africa, PNG | IDA | 0%, ***50%*** | Marginal effect on years to 1% mf |
| Irvine et al. (2018) | PNG | DA | 75-90% (data) | Fewer MDA rounds required (up to 10+) |
| Smith et al. (2020) | Africa, PNG | IA | 0-80% (data) | Elimination probability higher if VC |

**Table II:** PRIME-NTD summary table [26].

| **Principle** | **What has been done to satisfy the principle?** | **Where in the manuscript is this described?** |
| --- | --- | --- |
| 1. Stakeholder engagement | This study was prompted by questions from stakeholders, including the WHO, about the impact of vector control and the modelling consensus. | PRIME-NTD table, Supplementary. |
| 2. Complete modelling documentation | References have been provided to model development and validation.  The model code will be made publicly available on GitHub following publication. | Methods and References.  <https://github.com/emmalouisedavis/VectorControlReviewLF> |
| 3. Complete description of data used | No specific data was used in this study. Parameter values are given in the Methods and a table in the Supplementary Information. | Methods and Supplementary. |
| 4. Communicating uncertainty | Figure 1: Boxplots, including inter-quartile range (IQR) and outliers.  Figure 2: 95% confidence intervals presented as shaded regions.  Figure 3: Presented outcome as a probability. | In the Results and Figures. |
| 5. Testable model outcomes | Model outcomes were compared to previous modelling and field evidence and key findings were summarised in Conclusions. | In section: Review of existing evidence. |

**Table III:** Parameter values used in TRANSFIL simulations, taken from previously published model documentation if not otherwise stated [4,17,18].

| **Parameter** | **Value/Scenario** |
| --- | --- |
| Baseline mf prevalence (%) | 10% (±1%) |
| Drug | IA |
| MDA frequency | Annual |
| MDA coverage | 65% |
| MDA systematic non-adherence correlation | 0, 0.35, 0.7 |
| EPHP threshold (mf prevalence) | 1% |
| Primary vector species | Anopheles |
| Bite risk aggregation parameter, ($k$) | 0.01 – 0.1 |
| Annual biting rate (ABR) | 0 - 1200 |
| Vector control coverage | 0, 0.5, 0.8 |
| Insecticidal decay half-life | 2 years [24] |
| Bite rate per mosquito per month ($\lambda$) | 10 |
| Proportion of mosquitoes infected by infectious bite | 0.37 |
| L3 uptake and development parameter ($\kappa$) | 4.395 |
| L3 uptake and development parameter ($r_{1}$) | 0.055 |
| Mosquito death rate per month | 5 |
| Mf birth rate per female worm per month ($\alpha$) | 1 |
| Proportion L3 leaving mosquito per bite ($\psi_{1}$) | 0.414 |
| Proportion L3 leaving mosquito that enter host ($\psi_{2}$) | 0.32 |
| Proportion L3 entering host that develop to adults ($s_{2}$) | 0.00275 |
| Adult worm death rate per month ($\mu$) | 0.0104 |
| Mf death rate per month ($\gamma$) | 0.1 |
| Host death rate per month ($\tau$) | 0.00167 |
| Proportion of mf killed by IA treatment | 0.99 |
| Proportion of adult worms killed by IA treatment | 0.35 |
| Length of worm sterilisation after IA treatment (months) | 9 |
| Reduction in individual bite risk in presence of LLINs (efficacy) | 0.97 |

**Waning insecticide assumptions**

Assuming a 2-year half-life [24] of LLIN efficacy gives the following equation governing LLIN efficacy, interpreted as the reduction in individual bite risk when sleeping under an LLIN, for time, $t$, in years:

$Eff=0.97e^{-t/2}$.
